# Tracing the Volatilomic Fingerprint of the Most Popular Italian Fortified Wines

**DOI:** 10.3390/foods12102058

**Published:** 2023-05-19

**Authors:** Gonçalo Jasmins, Rosa Perestrelo, Jean Daniel Coïsson, Patrícia Sousa, José A. Teixeira, Matteo Bordiga, José S. Câmara

**Affiliations:** 1 CQM—Centro de Química da Madeira, Universidade da Madeira, Campus da Penteada, 9020-105 Funchal, Portugal; goncalo.jasmins@staff.uma.pt (G.J.); rmp@staff.uma.pt (R.P.); 2058017@student.uma.pt (P.S.); 2 Department of Pharmaceutical Sciences, Università degli Studi del Piemonte Orientale “A. Avogadro”, Largo Donegani 2, 28100 Novara, Italy; jeandaniel.coisson@uniupo.it (J.D.C.); matteo.bordiga@uniupo.it (M.B.); 3 CEB—Centre of Biological Engineering, University of Minho, Campus Gualtar, 4710-057 Braga, Portugal; jateixeira@deb.uminho.pt; 4 LABBELS-Associate Laboratory, University of Minho, Campus Gualtar, 4710-057 Braga, Portugal; 5 Departamento de Química, Faculdade de Ciências Exatas e Engenharia, Universidade da Madeira, Campus da Penteada, 9020-105 Funchal, Portugal

**Keywords:** volatilomic fingerprint, fortified wine, HS-SPME/GC-MS, molecular biomarkers

## Abstract

The aim of the current study was to provide a useful platform to identify characteristic molecular markers related to the authenticity of Italian fortified wines. For this purpose, the volatilomic fingerprint of the most popular Italian fortified wines was established using headspace solid-phase microextraction combined with gas chromatography–mass spectrometry (HS-SPME/GC-MS). Several volatile organic compounds (VOCs), belonging with distinct chemical groups, were identified, ten of which are common to all the analyzed fortified Italian wines. Terpenoids were the most abundant chemical group in Campari bitter wines due to limonene’s high contribution to the total volatilomic fingerprint, whereas for Marsala wines, alcohols and esters were the most predominant chemical groups. The fortified Italian wines VOCs network demonstrated that the furanic compounds 2-furfural, ethyl furoate, and 5-methyl-2-furfural, constitute potential molecular markers of Marsala wines, while the terpenoids nerol, α-terpeniol, limonene, and menthone isomers, are characteristic of Vermouth wines. In addition, butanediol was detected only in Barolo wines, and β-phellandrene and β-myrcene only in Campari wines. The obtained data reveal an adequate tool to establish the authenticity and genuineness of Italian fortified wines, and at the same time constitute a valuable contribution to identify potential cases of fraud or adulteration to which they are subject, due to the high commercial value associated with these wines. In addition, they contribute to the deepening of scientific knowledge that supports its valorization and guarantee of quality and safety for consumers.

## 1. Introduction

Due to the elaborate and accurate production processes, including the extraordinarily articulated conservation and ageing steps, wine experts consider fortified wines, which are typically produced in Europe, a high-added value and differentiated product. These wines are known to be of outstanding quality and have a substantial economic influence on the wine industry [1]. Fortified wines are distinguished by their high alcohol content (between 15 and 22%, *v*/*v*), due the addition of distilled spirits, typically grape spirit, and created under oxidative circumstances, which determines the fortified wine’s unique flavor and aroma profile [1].

Although the production of fortified wines is currently expanding globally, historically they are a product originates from Europe [2]. Some European countries continue to be the main producers despite the geographical expansion of their production [3]. Among the fortified wines, the most popular and well-known worldwide are Porto and Madeira wines from Portugal, Sherry wine from Spain, and Marsala wine from Italy.

As mentioned, the most renowned fortified wine in Italy is the Marsala wine, from Italy’s sunny southern region of Sicily. Vermouth is also produced in Italy following a great tradition in the Piedmont region [2]. Because they are flavored with unique blends of herbs and spices, vermouth constitutes a distinct type of fortified wine.

One of the major elements affecting wine quality is its aroma profile, which is mostly obtained from the grapes, fermentation, and ageing processes. Fortified wines, even more so, thanks to additional flavorings such as herbs, spices, and fruits, have attracted global attention. Characterizing their aromatic profile is important to determine their quality and define their complex structure.

Only the Marsala PDO region in Italy produces this Italian wine (the western part of Sicily Island). Catarratto, Damaschino, and Grillo cultivars (white grapes) are used to make Marsala (15–20% alcohol by volume), although red grapes can also be utilized to generate ruby-colored wines (e.g., Pignatello, Perricone, or Calabrese) [4,5]. Despite being typically vinified as a still wine, Marsala undergoes extensive pressing, resulting in a higher concentration of dry extract (25–30 g/L) and oxidizable materials [5]. The fermentation typically takes place between 18 and 20 °C, under regulated conditions. The fortification phase can be carried out either during or after fermentation, depending on the level of sweetness needed [4]. Neutral grape spirit, brandy, or mistelle can be used for fortification. In a process akin to solera, Marsala frequently ages in wooden barrels. According to the concentration of the reducing sugars, they are divided into three categories: “secco” (dry; less than 40 g/L), “semi-secco” (medium-dry; between 40 and 100 g/L), and “dolce” (sweet; more than 100 g/L) [6].

“Oro” (gold), “ambra” (amber), and “rubino” (ruby) are the three colors of Marsala, which have an average alcohol volume percentage of 18%. Depending on the sort of grapes used, the color differs. According to the level of ageing, this wine can be defined as: “fine” (over 1 year), “superiore” (over 2 years), “superior riserva” (over 4 years), “vergine” (over 5 years), and “stravecchio” (above 10 years) [6].

A specific kind of fortified wine (15–21% alcohol by volume) is called Vermouth. It is called “aromatized fortified wine,” which is made from grape-based wine by blending it with a variety of herbs and spices or by extracting their aromatic components [7]. Wormwood, also known as *Artemisia absinthium* L., is the primary flavoring component in vermouth, and the word “Vermouth” comes from the German word “Vermut”. The most frequently employed flavorings also include cloves, coriander, and chamomile. Vermouth was invented in the mid-seventeenth century, and industrial manufacturing of Vermouth began in Piedmont, southeast of Turin, Italy, in the late 18th century [8]. Today, Vermouth-style wines are widely consumed throughout Asia, Europe, and the United States, although most of them are assembled for commercial purposes in Poland and Russia [9].

Although more mature Vermouth wines exist, the maturing period typically lasts 5 years. While other varieties of Vermouth are also available, traditionally, Italian, and French variants are the most well liked internationally. The alcohol percentage in Italian Vermouths ranges from 15 to 17% by volume, while French Vermouths are dry and only reach about 18% alcohol by volume. Italian Vermouths are sweet. Vermouths typically have a bitter aftertaste and a pleasant, strong flavor because of the impact of the flavoring agents [9].

In 1860, in Italy, to compete with Vermouth, Mr. Gaspare Campari created the homonymous well-known bitter aperitif. The botanical recipe for Campari is different from that of Vermouth and consists of over 60 herbs and spices, the primary ones being cascarilla and cinchona bark, rhubarb stalks, bergamot essential oil, and ginseng roots [10]. The rich red color of Campari is obtained naturally from carmine cochineal, and the alcohol base is fortified white wine.

Barolo Chinato represents one of the most prestigious aromatized wines worldwide. This is part of the great centenary tradition of Piedmont, not only as an aromatized wine but also as a real tonic. Barolo Chinato was already produced at the end of the 19th century as a remedy for winter ailments. The idea of using it for therapeutic purposes came from Dr. Giuseppe Cappellano (pharmacist). Despite being prepared in the same way as classic vermouths, from which it derives as a concept, the two products are not even remotely comparable. The starting point is Barolo DOCG (controlled and guaranteed designation of origin) wine, to which sugar and alcohol are added, in the form of a cold infusion; a mix of alcohol and “drugs”, which is nothing more than a concentrate of spices, herbs, and medicinal roots also used in Vermouth. *Cinchona calisaya* is the tree from which the name derives, but rhubarb (*Rheum rhabarbarum*), cardamom (*Elettaria cardamomum*), and gentian (*Gentiana lutea*) are the most used aromatic and medicinal herbs used to produce this type of wine, being responsible for their most important flavours.

Nevertheless, the characterization of wine is quite complex, because volatile organic metabolites (VOMs) typically comprise distinct chemical groups such as acids, alcohols, aldehydes, esters, terpenes, phenols, and lactones, with a large range of polarities and concentrations. Analysis of these VOMs covers the process of extraction, desorption, separation, detection, and identification. High-performance liquid chromatography (HPLC) or gas chromatography combined with mass spectrometry (GC-MS) can be used to carry out the desorption, separation, and detection processes [11,12,13]. However, the accurate extraction of VOMs is critical for the whole determination, taking into consideration all the potential variables that could influence the extraction.

As a result, multiple extraction procedures have been applied to extract the VOMs from wine, which include steam distillation (SD), simultaneous distillation extraction (SDE) [14], stir bar extraction (SBSE) [15], and solid phase microextraction (SPME) [12,13]. However, the headspace solid-phase microextraction (HS-SPME) method has proven to be the most widely used method, due to its simplicity, accuracy, and speed [16]. The HS-SPME, first presented in 1990 by Arthur and Pawliszyn, is based on the ab/adsorption of the VOMs on a fiber, coated with a polymer, also recognized as the extraction phase. It consists, therefore, of the partitioning of the analyte between the extracting phase immobilized on a fused silica fiber and the headspace of the wine, which can take advantage of a favorable transfer of the VOMs into the latter [17].

Consequently, the capacity of the coupling with gas chromatography (GC) has been demonstrated to be a useful tool in identifying VOMs in a wide variety of foods, as well as for comparing the relative amounts of VOMs among samples when the same analytical procedure is used [18], due to its high separation effect on VOMs, strong identification ability, and to provide detailed information on the compounds [19].

Recently, HS-SPME combined with GC-MS has been successfully applied in several studies for the analysis of VOMs to determine the volatile flavor profiles of wine. Among them, Ivanova et al. [20] identified forty-four representative wine VOMs from eight varietal Macedonian and Hungarian wines through HS-SPME/GC-MS. Moreover, eighty-six aroma compounds were identified by Xiao et al. [21] using HS-SPME/GC-MS and an electronic nose, comprising five acids, thirty-four esters, ten alcohols, nine aldehydes, four ketones, four phenols, and ten nitrous and sulfuric compounds. This study evaluated the aroma compounds and determined the odor descriptors (OTs) for five typical Chinese liquors. In addition, Moreira et al. [22] quantified 38 carbonyl compounds (alkanals, alkenals, Strecker aldehydes, dialdehydes, ketones, and furan aldehydes) in Port wines using HS-SPME/GC-MS technology.

The principal aim of this work was to establish the volatilomic profile of fortified wines utilizing HS-SPME/GC-MS, to provide a platform to discover the characteristic molecular biomarkers that define the wine’s authenticity.

## 2. Materials and Methods

### 2.1. Reagents and Materials

Sodium chloride (NaCl, 99.5%) was purchased from Panreac (Barcelona, Spain), whereas 3-octanol (99%) used as an internal standard was obtained from Sigma-Aldrich (Madrid, Spain). The glass vials, fiber, and SPME holder for manual sampling were purchased from Supelco (Aldrich, Bellefonte, PA, USA). The SPME holder’s needle was coated with a fused silica fiber partially cross-linked with 50/30 µm divinylbenzene/carboxen/polydimethylsiloxane (DVB/CAR/PDMS).

### 2.2. Samples

In this study, nine different commercial Italian wines were analyzed. Barolo Chinato 2021 (Barolo): 18% alcohol by volume (ABV), production area Langhe, Piedmont; Campari^®^ 2021 (Campari): 25% ABV, production area Novi Ligure, Piedmont.

Marsala Vergine 2004 (Marsala2004): 19% ABV; Marsala Superiore Riserva 2007 (Marsala2007): 19% ABV; Marsala Medium Dry 2007 (MarsalaMD): 19% ABV; Marsala Superiore 2017 (MarsalaSup): 18% ABV. Production area of all the samples was Marsala, Sicily.

Vermouth white type 2021 (VermouthW): 18% ABV; Vermouth red type 2021 (VermouthR): 19% ABV; Vermouth Extra Dry white type 2021 (VermouthD): 18% ABV. The production area of all the samples was Cuneo, Piedmont. The abbreviations used for the analyzed samples are indicated in brackets.

### 2.3. HS-SPME Procedure

The fibers performance analysis, as well as the optimization of the experimental parameters (e.g., temperature, ionic strength, pH, extraction time), were not conducted, since previous studies performed in our laboratory had already disclosed the best parameters for wine analysis [14,15]. Briefly, 4 mL of wine, 12% NaCl and 5 µL of 3-octanol (concentration of 5 mg/L) was placed into a 20 mL amber glass vial. The vial was capped with a polytetrafluoroethylene (PTFE) septum and added into a thermostatic bath (40 ± 1 °C) with continuous magnetic stirring at 400 rpm. The fiber DVB/CAR/PDMS was introduced into the vial headspace and was left for 60 min to extract the analytes. This process was repeated in triplicate for each sample with independent aliquots. A blank was performed every day (10 min inside the injection port at 250 °C), before starting the first extraction, to ensure that no analytes from the previous days carried over.

### 2.4. Gas Chromatography Mass Spectrometry

The GC-MS analysis was conducted using an Agilent Technologies 6890N gas chromatography equipped with a Supelcowax^®^ 10 fused silica capillary column (60 m × 0.25 mm i.d. × 0.25 µm film thickness) provided by Supelco (Bellefonte, PA, USA) and interfaced with an Agilent 5975 quadrupole inert mass selective detector (Palo Alto, CA, USA). After HS-SPME extraction, the fiber was exposed in the injection port at 250 °C (equipped with a glass liner, 0.75 mm I.D.) for 7 min for the analyte’s desorption. The column flow rate of 1 mL/min (column-head pressure: 13 psi) was achieved by using helium (Air Liquide, Portugal, at a purity higher than 99%) as the carrier gas. The temperature of the oven was fixed as follows: 55 °C (1 min), a ramp of 1.50 °C/min to 100 °C (3 min), a ramp of 2 °C/min to 150 °C (4 min), a ramp of 5 °C/min to 200 °C, and this temperature was left for 10 min at the end. The total run time was 87 min. For the MS system, the temperatures of the transfer line, quadrupole, and ionization source were 250, 150, and 180 °C, respectively. The electron impact mass spectra were recorded at 70 eV, the ionization current was about 30 µA, and the acquisition mass range was set from *m/z* 30 to 300. The VOMs identification was assigned by comparison with the spectral data obtained with the data from the National Institute of Standards and Technology (NIST) MS 05 spectral mass libraries (Gaithersburg, MD, USA, NIST05) using the instrument data analysis program (G1701DA version D.02.00 by Agilent Technologies). The similarity threshold was then chosen for the spectral analysis and the VOM identification was higher than 80%. In addition, the VOMs were also identified by comparison with the applicable standards when available.

### 2.5. Statistical Analysis

The statistical analysis was processed by MetaboAnalyst 5.0, a web-based tool [23]. Prior to the statistical analysis, the data matrix was pre-processed to eliminate VOMs with missing values (MV), data transformation by cube root, and data scaling by auto-scaling.

A chemical network containing all the analytes identified in the GC-MS data was constructed using a network-building tool denominated Gephi (version 0.9.7). The data treatment was conducted through Excel, which consisted of two parts (nodes and edges), the nodes established the samples and the compound ID, which were then associated with each other through the edges, put differently, the edges formed the connection between the samples and compounds. The edge type (directed) and its weight (one) were left on default. After the data treatment in Excel, it was inserted into Gephi. The network layout used was “ForceAtlas 2” [24]. After that, a principal component analysis (PCA), as an investigational data analysis approach, was applied to analyze the group trends, and a PLS-DA was carried out to establish the discrimination among the fortified wines analyzed. All the VOMs with variable importance in projection (VIPs) values higher than 1.4 were recognized as potential molecular biomarkers.

## 3. Results and Discussions

### 3.1. Volatilomic Fingerprint of the Italian Fortified Wines

**Figure 1:**
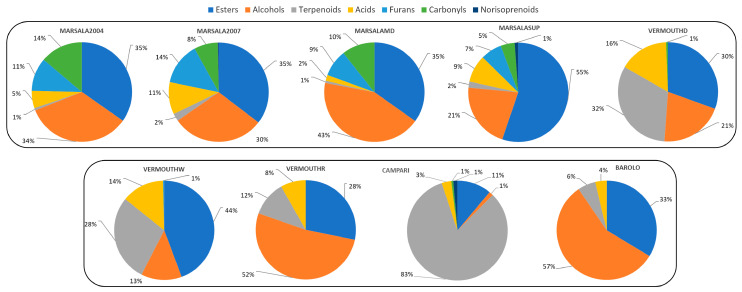
Contribution of each chemical group to the total volatilomic fingerprint of all the fortified wines analyzed.

Aroma is a crucial quality criterion for fortified wines. Thus, providing a platform to identify characteristic molecular biomarkers that define the wine’s authenticity is a key factor. A total of 56 VOMs were identified in fortified wines belonging to different chemical groups (Table S1, Supplementary Materials), such as terpenoids (19), esters (14), alcohols (7), carbonyl compounds (4), furanic compounds (4), acids (3), norisoprenoids (2), and 3 other VOMs. Figure 1 displays the distribution of VOMs according to their chemical groups, where it indicates that esters and alcohols were the most predominant chemical groups identified in the fortified wines studied, excluding Campari wines, since terpenoids have a high contribution of 83% to the total volatilomic fingerprint.

**Figure 2:**
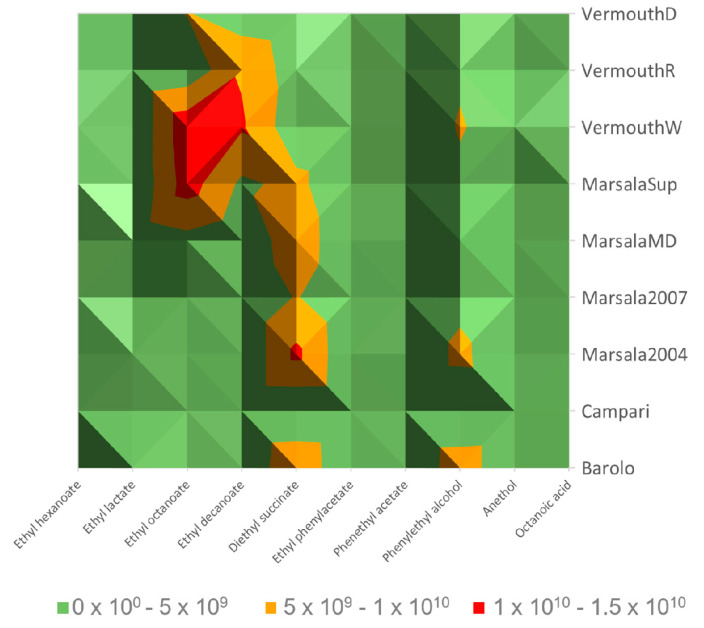
Surface map of the volatile organic metabolites (VOMs) common to all the fortified wines analyzed.

**Figure 3:**
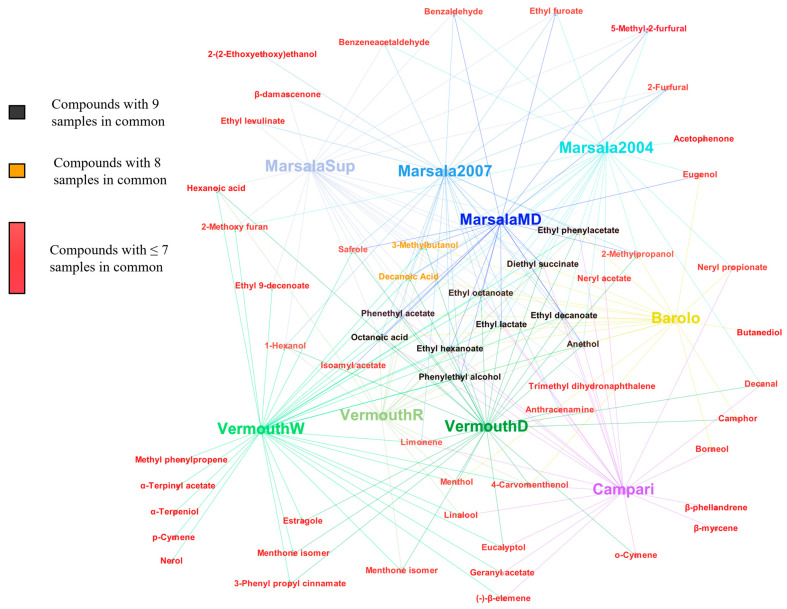
Aroma network of the fortified wines studied.

Among all VOMs identified, only 10 (ethyl hexanoate, ethyl lactate, ethyl octanoate, ethyl decanoate, diethyl succinate, ethyl phenylacetate, phenythyl acetate, phenylethyl alcohol, anethol, and octanoic acid) were common to all the Italian fortified wines analyzed, however, their contribution to the total volatilomic fingerprint was different for all the wines analyzed (Figure 2). Moreover, as can be seen in Figure 3, these VOMs are centralized in the middle of the aroma network of all the fortified wines analyzed.

Esters are qualitatively the second chemical group identified in fortified wines, and their contribution to the total volatilomic fingerprint ranged from 11 (Campari wines) to 55% (Marsala Superiore 2017). The contribution of this chemical family to Marsala superior is quite like Vermouth red type (VermouthR, 52%). On the other hand, the contribution by esters to the total volatilomic fingerprint in Marsala 2004 (35%), Marsala 2007 (35%), Marsala medium dry (MarsalaMD, 35%), Barolo (33%), and Vermouth extra dry (VermouthD, 30%) are quite similar. Moreover, esters presented a positive contribution to the overall wine aroma with fruit and floral odor notes since their OT is extremely low (a few µg/L) [25]. Ethyl hexanoate, ethyl octanoate, ethyl decanoate, and diethyl succinate were, on average, the most abundant esters identified in the fortified wines studied, and their contribution can be observed in Figure 2. On the other hand, 3-phenyl propyl cinnamate was only detected in VermouthW, ethyl levulinate in Marsala 2007 and Marsala Superiore 2017, and geranyl acetate in Campari and VermouthW fortified wines. Esters are generated enzymatically during yeast fermentation, whereas acetates are the result of the reaction between acetyl-CoA with alcohols that are produced from the degradation of amino acids or carbohydrates [26].

Alcohols are produced through yeast metabolism during the fermentation process by one of two pathways connected to amino acid metabolism: (1) the catabolism of the grape’s amino acids, the Ehrlich pathway, and/or (2) the production of α-keto acids during amino acid biosynthesis from sugar, the anabolic pathway [27]. The contribution of alcohols to the wine aroma depends on its concentration, if they are present at concentrations less than 300 mg/L they influence positively with fruit and flowers odors, while a negative aromatic contribution is likely when the wine comprises of an alcohol concentration above 400 mg/L [28]. On average, the highest total volatilomic fingerprint of alcohols was determined in Barolo wines (57%), followed by VermouthR (52%), MarsalaMD (43%), Marsala 2004 (34%), VermouthD (32%), Marsala 2007 (30%), MarsalaSup ~ VermouthD (21%), and VermouthW (13%). The highest alcohol contribution to the total volatilomic fingerprint of Barolo, VermouthD, and MarsalaMD is mainly explained by the highest GC peak area of 3-methylbutanol and 2-phenylethanol. These alcohols are already identified in Marsala [29] and Madeira wines and their presence was linked positively to aromas such as fruits (e.g., banana) and flowers (e.g., rose) [13]. On the other hand, 3-methylbutanol was not identified in Campari wine, whereas 2-(2-ethoxyethanol) and butanediol were only detected in Marsala2007 and Barolo wines, receptively, therefore it could be considered a characteristic molecular biomarker of these fortified wines, see Figure 3.

Regarding the acids, like hexanoic acid and octanoic acid, present in the wines, they were classified as unpleasant aromas (e.g., fatty, sweaty, rancid, cheese) at a concentration above 20 mg/L, however, they are crucial VOMs for flavor quality since they impart pleasant aromas (e.g., woody, brandy, almond) to wine at appropriate concentration levels [30]. This chemical group is formed during alcoholic fermentation. This chemical group showed a different contribution to the total volatilomic fingerprint, being the highest in Vermouth wines (on average, 13%), followed by Marsala (6.8%), Barolo (4%), and Campari (3%). Octanoic acid was detected in all the fortified wines analyzed, whereas decanoic acid was not detected in Campari wine. Furthermore, hexanoic acid was detected in all the Vermouth and MarsalaSup wines.

Although the VOMs (e.g., alcohols, esters, acids) formed during the fermentation process are typically the utmost significant contributors to the base aroma of the fortified wine, the varietal VOMs (e.g., terpenoids) biosynthesized during grape evolution and ripening also represent a vital element of the characteristics of many wines. In this sense, terpenoids are an exceptional group of aromatic VOMs from a varietal origin, which can be utilized as a potential tool to ensure the authenticity and typicity of wines. Campari seems to be the richest in terpenoids (83%) in contrast to Marsala wines, which have the lowest percentage since the contribution of its terpenoids to the total volatilomic fingerprint was lower than 2%. Limonene is the most abundant, in terms of the GC peak area, terpenoid identified in Campari wines. Limonene is described as a positive varietal odor descriptor in the wines, such as lemon and orange, and from a healthy point of view, the chemopreventive and chemotherapeutic characteristics of limonene against human cancers have been extensively proved by Paduch et al. [31]. In VermouthR and VermouthD, limonene and menthol were the predominant terpenoids identified, whereas in VermouthW estragole, α-terpinyl acetate, and anethol were the most abundant. Moreover, β-myrcene and β-phellandrene were only detected in Campari wine, which could be characteristic molecular biomarkers that define Campari’s authenticity, see Figure 3. In addition, α-terpeniol, α-terpenyl acetate, nerol, methyl phenylpropene, and p-cymene could also be characteristic molecular biomarkers of VermouthW.

Furanic compounds, formed through three pathways, namely (1) pyrolysis of carbohydrates, (2) dehydration of sugars by the Maillard reaction, and (3) caramelization, were not identified in Barolo, Campari, VermouthR, and VermouthD, whereas 2-furfural, 5-methyl-2-furfural, and ethyl furoate were identified in all analyzed Marsala wines. In addition, 2-methoxy furan was identified in Marsala 2004, Marsala Superiore 2017, and VermouthW. As can be observed in Figure 3, furanic compounds were more correlated with Marsala wines and for this reason, they could be used as potential markers of these wines. From a sensorial point of view, their contribution to the wine aroma is not predictably outstanding due to their high OTs [25].

Carbonyl compounds contributed 14% and 11% for the total volatilomic fingerprint of Marsala2004 and MarsalaMD, respectively. The lowest contribution was verified in Campari and VermouthD wines (<1%), followed by MarsalaSup (5%), and Marsala2007 (8%). Decanal was detected in Campari, Marsala2004, and VermouthD, whereas acetophenone was identified in Marsala2004. Benzaldehyde was predominant in the Marsala wines studied.

### 3.2. Multivariate Analysis

The GC peak area of 56 VOMs (GC-MS data set) identified in the Italian fortified wines (samples) were normalized and submitted to PCA and PLS-DA to identify the main sources of variability and to characterize the fortified wines according to their volatilomic fingerprint.

**Figure 4:**
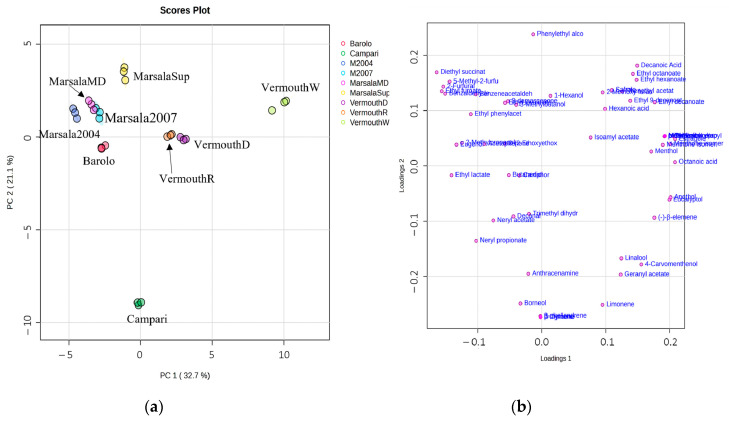
PCA of the volatilomic fingerprint of the Italian fortified wines (*n* = 3 for each data point): (**a**) score scatter plot and (**b**) loading weight plot.

Figure 4 shows the PCA score plot and the loading weight plot of the two first principal components (PC1 vs PC2), which explains 53.8% of the total variability in the data set.

The Campari and Barolo wines were positioned in PC1 and PC2 negative, which is mainly due to the presence of borneol, β-phellandrene, β-myrcene, ethyl lactate, butanediol, and camphor, whereas VermouthW (PC1 and PC2 positive) was essentially characterized by menthol, estragole, and the menthone isomer. Marsala wines (Marsala2004, Marsala2007, MarsalaSup, MarsalaMD) displayed in PC1 negative and PC2 positive were mainly associated with 5-methyl-2-furfural, 2-furfural, ethyl furoate, benzaldehyde, benzene acetaldehyde, and diethyl succinate.

**Figure 5:**
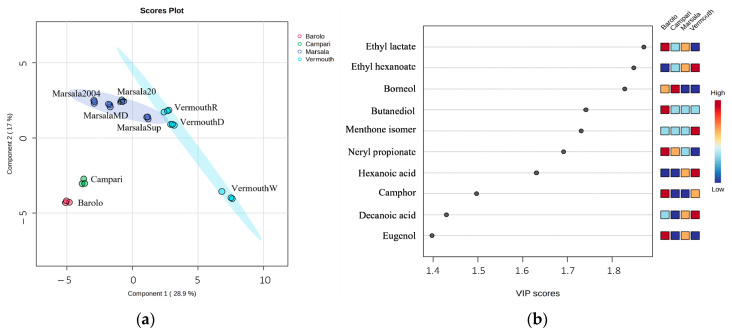
PLS-DA of the total volatilomic fingerprint of the Italian fortified wines (*n* = 3 for each data point): (**a**) score scatter plot and (**b**) VIP scores.

To further understand the differences between the Italian fortified wines, the GC-MS data set was submitted to PLS-DA, and a clear discrimination was observed (Figure 5a). Combining the VIP values higher than 1 (Figure 5b), 10 VOMs were selected as putative characteristic molecular biomarkers for the studied Italian fortified wines. These characteristic molecular biomarkers included ethyl lactate, ethyl hexanoate, borneol, butanediol, menthone, neryl propionate, hexanoic acid, camphor, decanoic acid, and eugenol.

**Figure 6:**
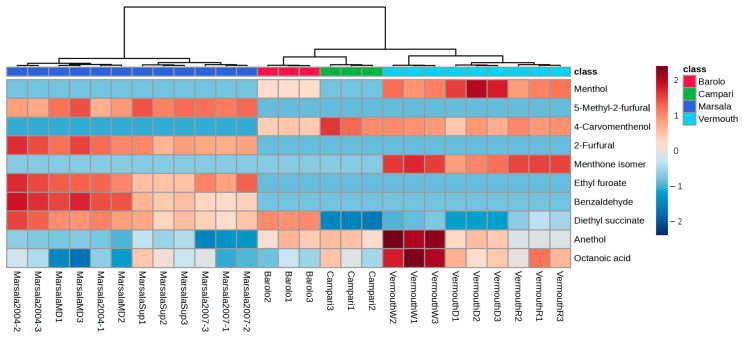
HCA and heatmap of the putative characteristic molecular biomarkers identified in Italian fortified wines generated by the average algorithm and Pearson distance analysis.

Hierarchical cluster analysis (HCA) was performed to understand the relationships between the analyzed Italian fortified wines. As can be seen in Figure 6, the Marsala wines were grouped in one cluster, while Campari and Barolo were grouped in another cluster, and Vermouth wines in a different cluster. However, Campari, Barolo, and Vermouth wines share the same upper clusters. In addition, the observed clusters can be supported by the similarity/difference of the GC peak area of the identified VOMs. These results were supported by the data obtained by PCA and PLS-DA, as the GC peak area of the VOMs was different between the analyzed wines. The VOMs recognized as potential molecular biomarkers of Italian fortified wines result from the fermentation process other than the raw material.

## 4. Conclusions

A total of 56 VOMs, belonging to distinct chemical groups, such as 19 terpenoids, 14 esters, 7 alcohols, 4 carbonyl compounds, 4 furanic compounds, 3 acids, 2 norisoprenoids, and 3 other VOMs were identified using HS-SPME/GC-MS. Among the VOMs identified in Italian fortified wines, only seven esters, one alcohol, one terpenoid, and one acid were common to all, but their contribution to the total volatilomic fingerprint was different. Terpenoids were the most abundant chemical group in Campari (83% of the total volatilomic fingerprint), while in Marsala, esters were the most predominant (on average, 40%). From a sensorial point of view, terpenoids and esters contributed positively to the complexity of the overall aroma with fruit and floral odor descriptors, as their odor threshold is too low (a few µg/L).

According to the data available on the aroma network, nerol, α-terpeniol, limonene, menthone isomers were more indicative as characteristic molecular biomarkers of Vermouth wines, as well as 2-furfural, ethyl furoate, and 5-methyl-2-furfural for Marsala wines. Furthermore, butanediol was only detected in Barolo wines, whereas β-phellandrene and β-myrcene were only identified in Campari. The GC dataset submitted to PLS-DA analysis allowed for the discrimination of the Italian fortified wines, and 10 VOMs showed that VIP scores higher than 1 were responsible for this clear separation. These characteristic molecular biomarkers included ethyl lactate, ethyl hexanoate, borneol, butanediol, menthone isomer, neryl propionate, hexanoic acid, camphor, decanoic acid, and eugenol. The data obtained denotes an appropriate approach to establish their authenticity and genuineness, as a valuable contribution to identifying possible adulteration and, consequently, valorising the commercial value of Italian fortified wines. To conclude, future work should be carried out to better fundament the research data displayed in this paper, by analyzing more samples from different varieties of each of the main fortified Italian wine chemical groups.

## Data Availability

Data is contained within the article or Supplementary Materials.

## Supplementary Materials

The following supporting information can be downloaded at: https://www.mdpi.com/article/10.3390/foods12102058/s1, Table S1. Volatile organic metabolites (VOMs) identified in Italian fortified wines using HS-SPME/GC-MS; Figure S1. Typical HS-SPME/GC-MS volatilomic profile of Barolo wine; Figure S2. Typical HS-SPME/GC-MS volatilomic profile of Campari wine; Figure S3. Typical HS-SPME/GC-MS volatilomic profile of Marsala wines; Figure S4. Typical HS-SPME/GC-MS volatilomic profile of Vermouth wines.
