# Supplementary material for: Tracing the Volatilomic Fingerprint of the Most Popular Italian Fortified Wines
Source: Foods. 2023 May 19;12(10):2058. doi: 10.3390/foods12102058 (PMC10217346; doi:10.3390/foods12102058)
Supplement: Supplementary file 1 [file foods-12-02058-s001.zip › foods-2327274-supplementary.pdf]

Table S1. Volatile organic metabolites (VOMs) identified in Italian fortified wines using HS-SPME/GC-MS

RT (min)	Chemical families	Formula	GC Peak area × 10^8 ± standard deviation								
			**Barolo**	**Campari**	**Marsala2004**	**Marsala2007**	**MarsalaMD**	**MarsalaSup**	**VermouthW**	**VermouthR**	**VermouthD**
	**Esters**										
24.29	Isoamyl acetate	$C_7H_{14}O_2$	-	-	-	-	2.45±0.36	-	1.61±0.12	6.38±0.43	-
33.81	Ethyl hexanoate	$C_8H_{16}O_2$	0.65±0.02	0.85±0.01	1.31±0.12	7.23±0.91	2.42±0.24	15.1±2.18	15.6±2.76	10.9±1.17	10.8±0.97
43.88	Ethyl lactate	$C_5H_{10}O_3$	48.0±8.71	2.95±0.35	12.0±0.96	0.60±0.01	14.7±1.99	1.21±0.11	0.28±0.02	0.31±0.02	0.18±0.01
50.95	Ethyl octanoate	$C_{10}H_{20}O$	7.64±0.08	1.95±0.05	3.50±0.29	6.51±0.18	34.6±5.77	124±17.5	135±24.07	13.9±1.48	52.2±6.98
60.63	3-Phenyl propyl cinnamate	$C_{10}H_{18}$	-	-	-	-	-	-	3.13±0.61	-	0.17±0.03
64.29	Ethyl levulinate	$C_7H_{12}O_3$	-	-	-	0.91±0.13	-	1.22±0.14	-	-	-
65.67	Ethyl decanoate	$C_{12}H_{24}O$	10.2±1.81	1.31±0.12	1.56±0.20	1.62±0.22	9.27±0.59	40.5±4.11	106±12.3	95.3±4.33	19.1±1.49
68.06	Diethyl succinate	$C_8H_{14}O_4$	88.7±1.76	4.83±0.74	112±17.2	53.6±6.52	84.1±4.44	63.8±11.9	14.1±1.97	22.3±4.26	8.69±0.01
68.80	Ethyl 9-decenoate	$C_{12}H_{22}O$	-	-	-	-	-	3.61±0.40	4.28±0.77	-	-
70.60	Neryl acetate	$C_{12}H_{20}O$	-	2.11±0.09	2.02±0.38	-	-	1.78±0.23	-	-	-
71.95	Geranyl acetate	$C_{12}H_{20}O$	-	6.71±0.21	-	-	-	-	4.67±0.19	-	-
72.84	Neryl propionate	$C_{13}H_{22}O$	1.79±0.01	1.34±0.16	1.98±0.25	-	-	-	-	-	-
73.72	Ethyl phenylacetate	$C_{10}H_{12}O$	5.71±1.01	1.42±0.12	5.19±1.01	1.87±0.34	4.27±0.43	2.46±0.46	1.12±1.16	1.26±0.15	1.14±0.11
74.95	Phenethyl acetate	$C_{10}H_{12}O$	6.21±0.87	0.30±0.06	3.9±0.51	1.21±0.13	3.09±0.56	1.53±0.29	10.9±0.96	9.78±1.33	5.92±0.55
	**Alcohol**										
23.01	2-Methylpropanol	$C_4H_{10}O$	16.4±3.08	-	3.75±0.54	0.37±0.03	6.89±0.77	-	-	-	1.14±0.13
31.52	3-Methylbutanol	$C_5H_{12}O$	163±11.1	-	71.5±7.11	24.3±0.55	134±19.9	23.2±3.53	33.6±1.62	252±29.1	27.7±2.88
43.99	1-Hexanol	$C_6H_{14}O$	-	-	-	1.42±0.14	-	35.8±4.02	0.81±0.08	1.03±0.05	2.78±0.17
58.83	Butanediol	$C_4H_{10}O_2$	24.6±4.20	-	-	-	-	-	-	-	-
64.75	2-(2-Ethoxyethoxy)ethanol	$C_6H_{14}O_3$	-	-	-	0.68±0.11	-	-	-	-	-
77.99	Eugenol	$C_{10}H_{12}O$	3.63±0.55	-	2.58±0.18	-	1.81±0.28	-	-	-	-
78.26	Phenylethyl alcohol	$C_8H_{10}O$	77.2±4.12	2.89±0.12	63.0±8.16	35.2±5.83	48.6±8.67	39.5±2.35	53.9±4.98	43.5±1.3	34.7±3.45
	**Terpenoids**										
27.35	β-myrcene	$C_{10}H_{16}$	-	2.60±0.21	-	-	-	-	-	-	-
29.63	Limonene	$C_{10}H_{16}$	-	130±18.4	-	1.08±0.06	-	-	8.71±0.33	16.4±1.43	11.9±0.23
31.83	β-phellandrene	$C_{10}H_{16}$	-	1.49±0.29	-	-	-	-	-	-	-
32.14	Eucalyptol	$C_{10}H_{18}O$	-	3.27±0.21	-	-	-	-	11.2±0.74	-	7.15±1.09
37.60	o-Cymene	$C_{10}H_{14}$	-	7.66±0.22	-	-	-	-	-	-	-
37.91	p-Cymene	$C_{10}H_{14}$	-	-	-	-	-	-	2.21±0.31	-	-
52.38	α-Terpeniol	$C_{12}H_{22}O$	-	-	-	-	-	-	8.11±1.32	-	-
54.04	Menthone isomer	$C_{10}H_{18}O$	-	-	-	-	-	-	8.33±0.44	7.81±0.04	5.56±0.71
56.21	Menthone isomer	$C_{10}H_{18}O$	-	-	-	-	-	-	3.69±0.17	-	3.75±0.58
56.52	Nerol	$C_{10}H_{18}O$	-	-	-	-	-	-	2.03±0.39	-	-
58.21	Camphor	$C_{10}H_{16}O$	8.47±0.44	-	-	-	-	-	-	-	0.97±0.01
58.88	Linalool	$C_{10}H_{18}O$	-	12.1±0.65	-	-	-	-	7.11±1.21	12.8±1.46	-
63.69	4-Carvomenthenol	$C_{10}H_{18}O$	5.92±0.18	11.2±2.21	-	-	-	-	8.74±0.33	8.88±0.49	7.27±1.29

65.90	Menthol	$C_{10}H_{18}O$	5.69±0.08	-	-	-	-	-	14.4±1.82	13.8±1.85	25.5±4.25
68.30	Estragole	$C_{10}H_{12}O$	-	-	-	-	-	-	59.6±7.63	-	23.2±2.71
69.57	α-Terpinyl acetate	$C_{12}H_{20}O$	-	-	-	-	-	-	29.0±3.01	-	-
69.74	Borneol	$C_{10}H_{18}O$	0.82±0.08	1.38±0.12	-	-	-	-	-	-	-
75.49	Anethol	$C_{10}H_{12}O$	8.04±1.37	8.18±0.96	3.16±0.2	1.62±0.17	2.88±0.37	4.1±0.49	23.3±1.88	5.35±0.12	8.13±0.81
77.29	Safrole	$C_{10}H_{10}O$	-	-	-	2.43±0.30	0.24±0.02	5.09±0.58	3.96±0.21	-	4.55±0.66
	**Acids**										
71.39	Hexanoic acid	$C_{16}H_{34}O$	-	-	-	-	2.58±0.17	4.08±0.80	1.85±0.01	6.08±0.91	6.66±0.05
75.90	Octanoic acid	$C_6H_{12}O_2$	5.69±0.45	6.62±0.94	5.89±0.38	5.47±0.89	4.33±0.33	6.82±0.69	11.2±0.71	7.86±1.22	7.5±0.53
82.81	Decanoic Acid	$C_8H_{16}O_2$	12.9±2.02	-	17.3±2.86	16.3±2.76	1.59±0.30	30.2±2.85	79.6±13.63	32.4±0.02	37.3±1.32
	**Furanic compounds**										
53.90	2-Furfural	$C_5H_4O_2$	-	-	39.3±5.75	23.6±0.79	34.0±6.04	25.3±3.65	-	-	-
62.00	5-Methyl-2-furfural	$C_6H_6O_2$	-	-	2.65±0.14	3.63±0.17	3.59±0.66	3.74±0.42	-	-	-
65.20	Ethyl furoate	$C_7H_8O_3$	-	-	1.61±0.23	1.19±0.21	1.33±0.15	0.75±0.01	-	-	-
66.63	2-Methoxy furan	C_7H_{14}	-	-	1.09±0.14	-	-	2.02±0.31	2.51±0.01	-	-
	**Carbonyl compounds**										
56.11	Decanal	$C_{10}H_{20}O$	-	1.39±0.20	3.88±0.09	-	-	-	-	-	2.01±0.34
58.68	Benzaldehyde	C_7H_6O	-	-	51.0±7.63	14.7±1.05	47.1±5.95	19.2±2.36	-	-	-
67.11	Benzeneacetaldehyde	C_8H_8O	-	-	1.00±0.12	1.30±0.14	-	1.71±0.24	-	-	-
67.53	Acetophenone	C_8H_8O	-	-	0.76±0.09	-	-	-	-	-	-
	**Norisoprenoids**										
75.23	β-damascenone	$C_{13}H_{18}O$	-	-	-	0.65±0.03	-	1.21±0.22	-	-	-
75.59	Trimethyl	$C_{13}H_{16}$	-	2.82±0.41	-	-	-	4.18±0.16	-	-	-
	**Others**										
51.87	Methyl phenylpropene	$C_{10}H_{12}$	-	-	-	-	-	-	2.71±0.31	-	-
55.24	Anthracenamine	$C_{14}H_{11}N$	-	1.67±0.16	-	1.28±0.15	-	-	-	-	0.47±0.01
62.16	(-)-β-elemene	$C_{10}H_{18}$	-	1.33±0.08	-	-	-	-	3.08±0.41	-	-

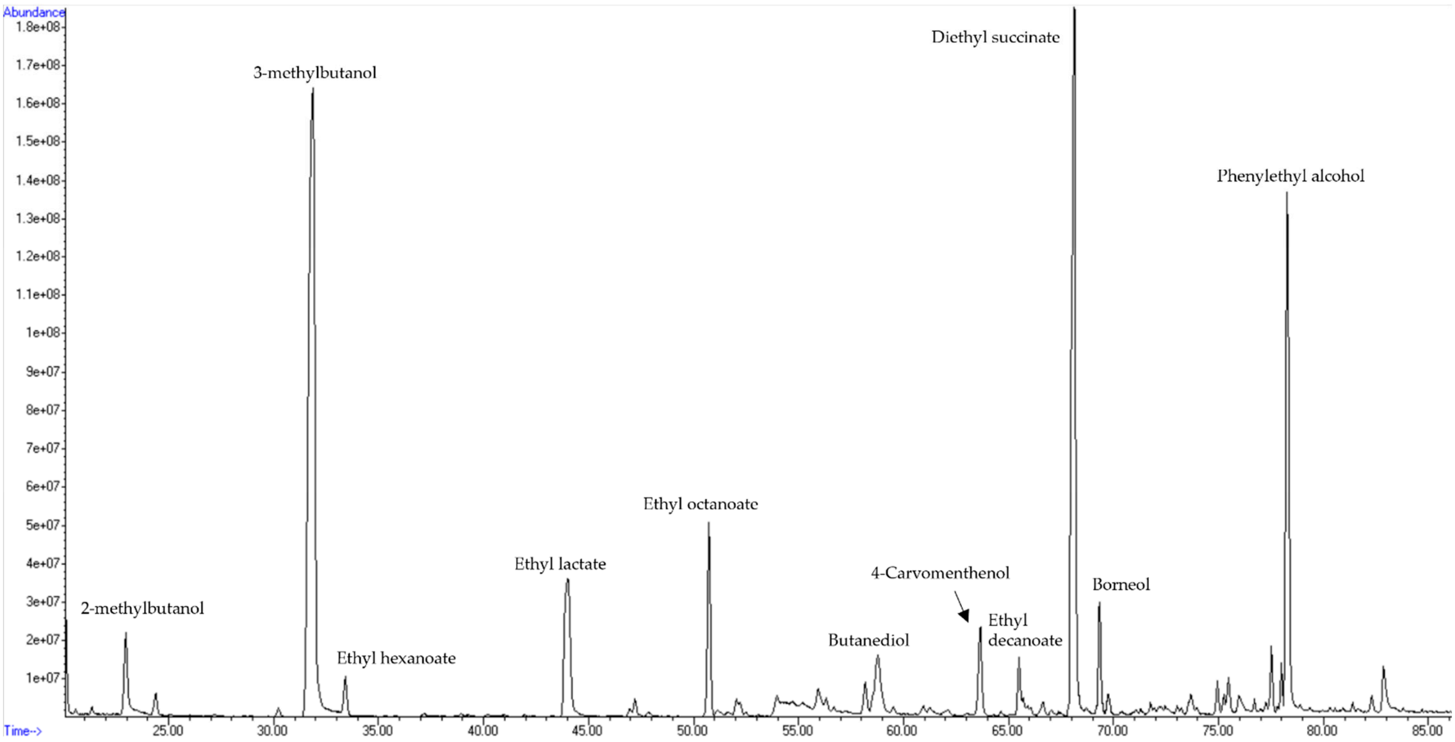

Figure S1. Typical HS-SPME/GC-MS volatilomic profile of Barolo wine

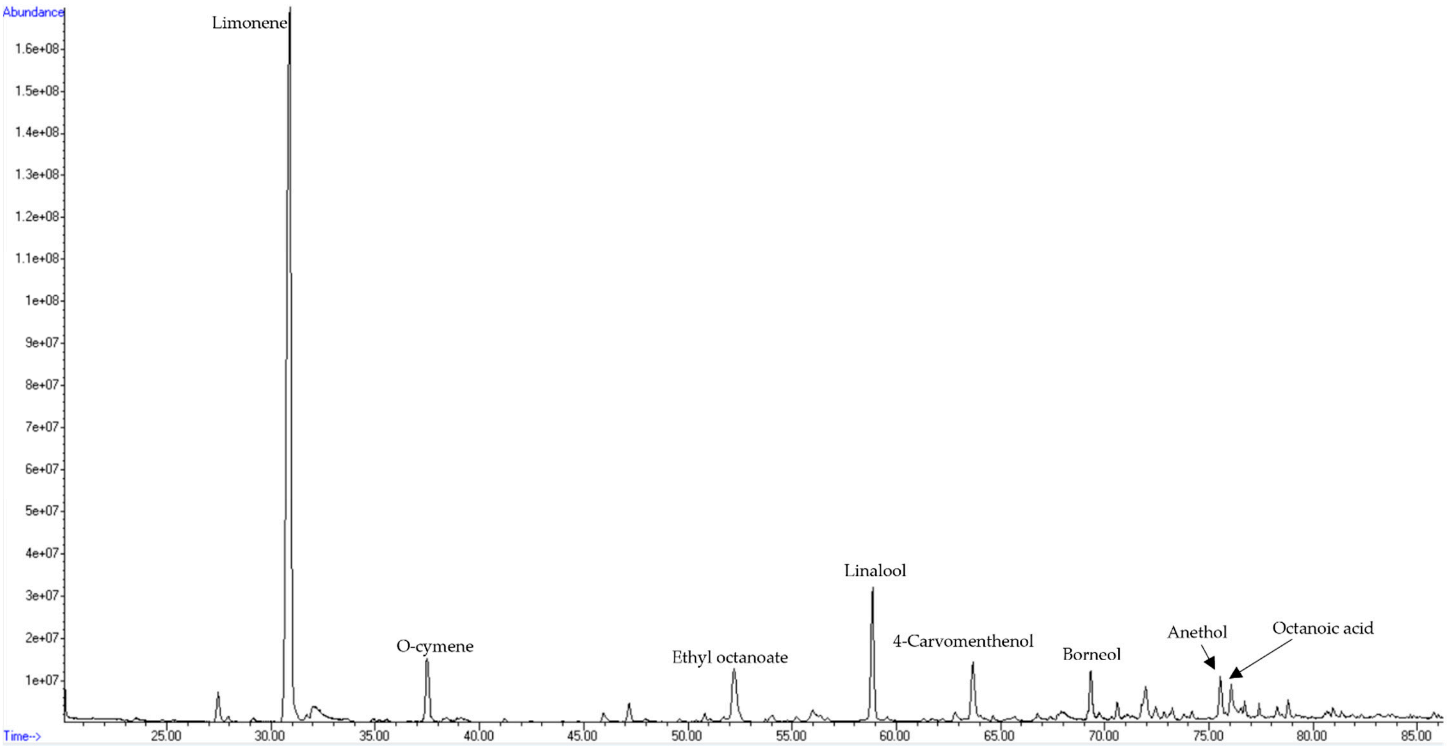

Figure S2. Typical HS-SPME/GC-MS volatilomic profile of Campari wine.

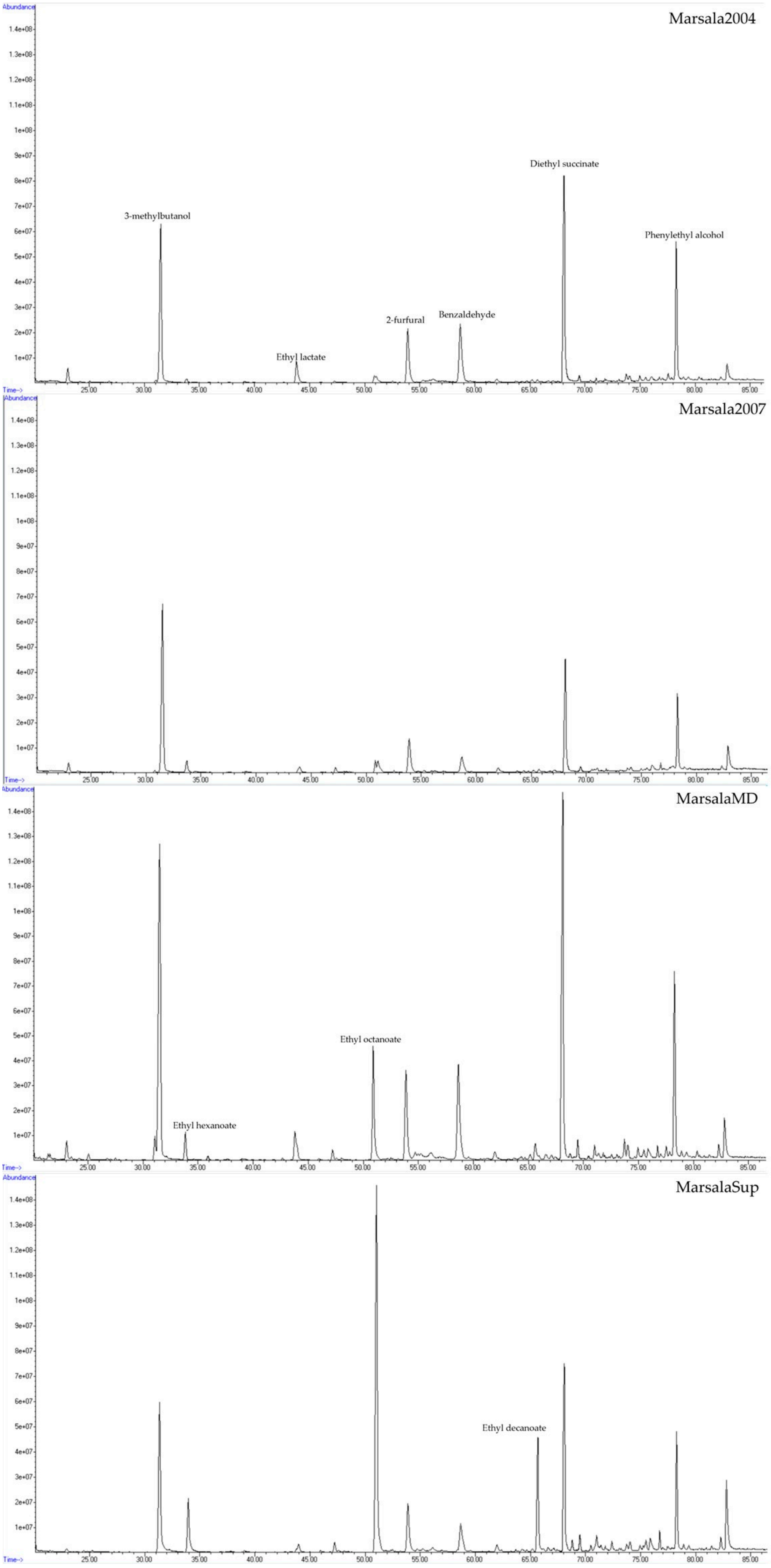

Figure S3. Typical HS-SPME/GC-MS volatilomic profile of Marsala wines.

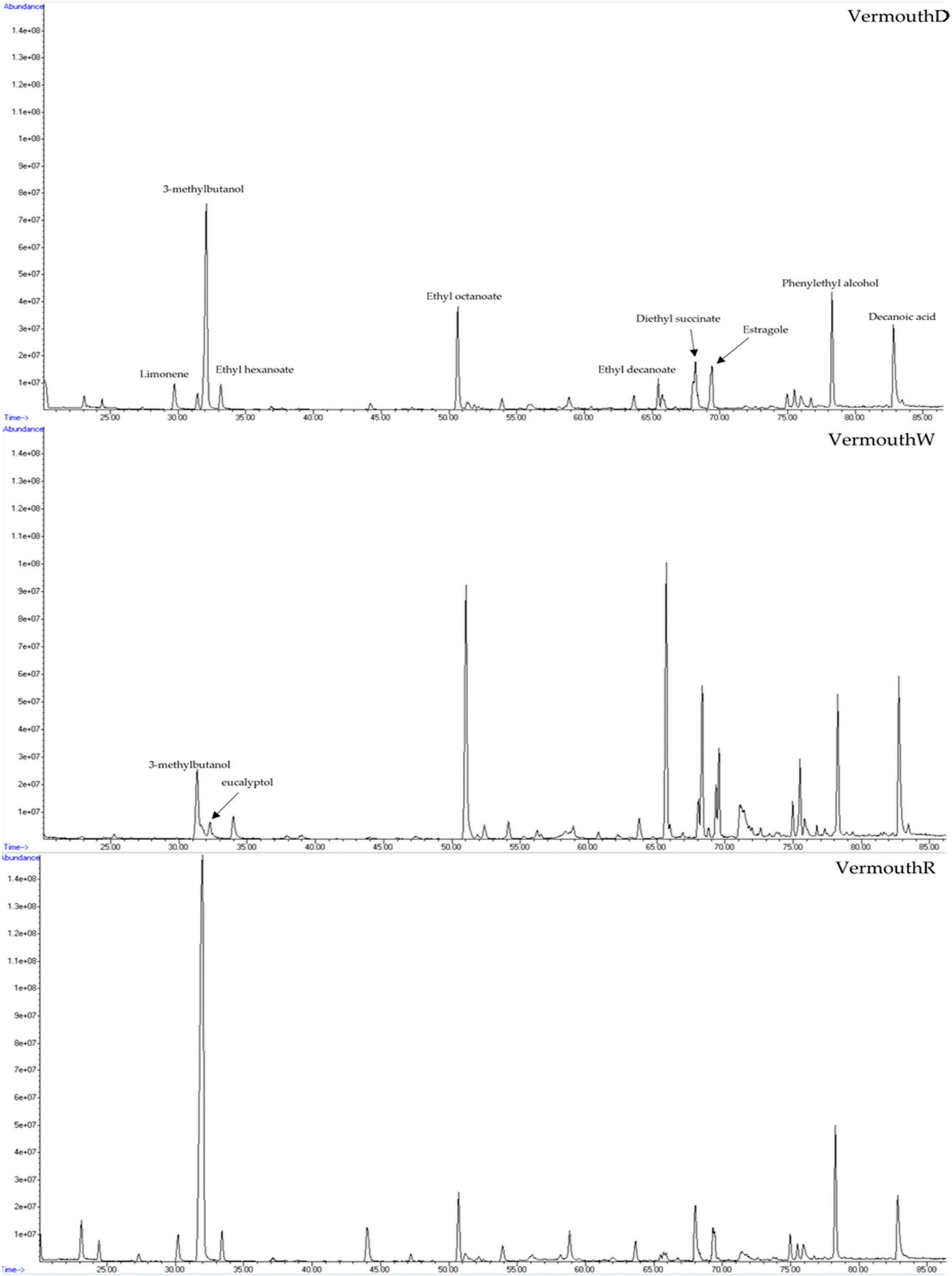

Figure S4. Typical HS-SPME/GC-MS volatilomic profile of Vermouth wines.
